# Complete Response to First-Line Trastuzumab Deruxtecan in Human Epidermal Growth Factor Receptor 2 (HER2)-Positive Extra-axial Central Nervous System Metastases

**DOI:** 10.7759/cureus.116819

**Published:** 2026-09-24

**Authors:** Maria João Oura, Rita Pinho, Fernando Osório, Miguel Barbosa, Ana Valente

**Affiliations:** 1 Medical Oncology, Unidade Local de Saúde (ULS) São João, Porto, PRT; 2 Surgery, Unidade Local de Saúde (ULS) São João, Porto, PRT

**Keywords:** antibody-drug conjugate, central nervous system metastasis, extra-axial metastasis, her2-positive breast cancer, leptomeningeal disease (lmd), trastuzumab-deruxtecan

## Abstract

Trastuzumab deruxtecan (T-DXd) is an antibody-drug conjugate approved for the treatment of unresectable or metastatic human epidermal growth factor receptor 2 (HER2)-positive breast cancer in patients who have received a prior anti-HER2-based regimen. HER2-positive breast cancer has a high propensity for central nervous system (CNS) spread, which confers significant morbidity and a poor prognosis. We report the case of a 47-year-old woman with metastatic HER2-positive breast cancer who developed debilitating symptomatic CNS metastases, predominantly extra-axial leptomeningeal lesions. Locoregional treatment options were not feasible, and the patient received off-label first-line systemic therapy with T-DXd. Rapid and marked neurological improvement was observed within weeks. Follow-up MRI at three months demonstrated complete radiologic resolution of all CNS lesions. As of April 2026, after 29 cycles of T-DXd, the patient remains clinically asymptomatic with no evidence of CNS disease on MRI, corresponding to a progression-free survival exceeding 20 months. This case highlights the potential role of T-DXd in symptomatic CNS metastases, particularly leptomeningeal disease, and underscores the need for prospective research into its intracranial efficacy in this distinct and challenging subset of patients.

## Introduction

Trastuzumab deruxtecan (T-DXd) is an antibody-drug conjugate (ADC) composed of a human epidermal growth factor receptor 2 (HER2)-directed monoclonal antibody linked to a topoisomerase I inhibitor payload via a tumor-selective cleavable linker. Based on the results of the pivotal phase III DESTINY-Breast03 trial, T-DXd is approved for patients with unresectable or metastatic HER2-positive breast cancer that progressed during or after treatment with trastuzumab and a taxane in the context of advanced or metastatic disease or within six months after (neo)adjuvant treatment [1].

HER2-positive breast cancer has one of the highest propensities for central nervous system (CNS) spread among all breast cancer subtypes, with approximately 30-50% of patients developing brain metastases during the course of disease [2]. CNS involvement confers a poor prognosis and significantly impairs quality of life. Management of CNS metastases remains challenging due to the selective permeability of the blood-brain barrier (BBB) and the historically limited efficacy of systemic agents in the intracranial compartment. Locoregional options, including surgery, stereotactic radiosurgery, and whole-brain radiotherapy, are not universally applicable, particularly in the context of diffuse, extra-axial, or leptomeningeal disease.

## Case presentation

The patient was a 47-year-old Caucasian woman with no relevant medical history. In August 2021, she was diagnosed with locally advanced, hormone receptor-negative, HER2-positive breast cancer after noticing a lump in her left breast. Imaging revealed a 27 × 18 mm lesion in the inferolateral quadrant and multiple axillary lymph node metastases (cT2N2M0). She received neoadjuvant therapy with the TCHP regimen (docetaxel 75 mg/m², carboplatin AUC 6, trastuzumab 8 mg/kg loading dose followed by 6 mg/kg maintenance, and pertuzumab 840 mg loading dose followed by 420 mg subsequent doses), administered every 21 days for six cycles. Left mastectomy and axillary lymph node dissection were performed in February 2022, demonstrating a pathological complete response. Postoperative radiotherapy was administered to the chest wall, followed by completion of adjuvant trastuzumab in September 2022, after which the patient entered surveillance.

In August 2023, she developed progressive gait imbalance. Brain MRI revealed a right cerebellar lesion measuring 30 × 21 × 12 mm, with no extracranial disease. Following multidisciplinary discussion, surgical resection was performed, and pathology confirmed metastatic breast carcinoma. Postoperative stereotactic radiotherapy was delivered to the surgical bed, followed by surveillance.

In June 2024, the patient presented with neurological deficits, including diplopia, right-sided facial paresis, gait ataxia, and dysphagia. Brain MRI revealed multiple extra-axial lesions with dural attachment in the bilateral inferior cerebellar tentorium, the left interhemispheric falx, and the posterior aspect of the petrous apex (largest measuring 25 × 13 × 15 mm), along with smaller lesions in the right anterior temporal lobe, the left optic chiasm, the left cerebellar hemisphere, and the right cerebral peduncle. The pattern was consistent with dural and leptomeningeal involvement. Systemic restaging confirmed CNS-limited metastatic disease with no extracranial disease.

Because the patient had symptomatic, CNS-limited metastatic disease with no feasible locoregional therapeutic options, off-label T-DXd (5.4 mg/kg every 21 days) was initiated in July 2024 as first-line systemic therapy. Neurological symptoms improved rapidly within the first weeks, with marked resolution of gait ataxia, diplopia, facial paresis, and dysphagia, restoring the patient's functional independence and quality of life. The first follow-up MRI, performed three months after treatment initiation, demonstrated complete radiologic resolution of all CNS lesions. Treatment was well tolerated, with no clinically significant toxicity, including interstitial lung disease (ILD)/pneumonitis, during regular clinical and radiological monitoring. Figure 1 illustrates this complete intracranial response, comparing the baseline brain MRI obtained in June 2024 with the first response assessment MRI performed in October 2024. 

**Figure 1:**
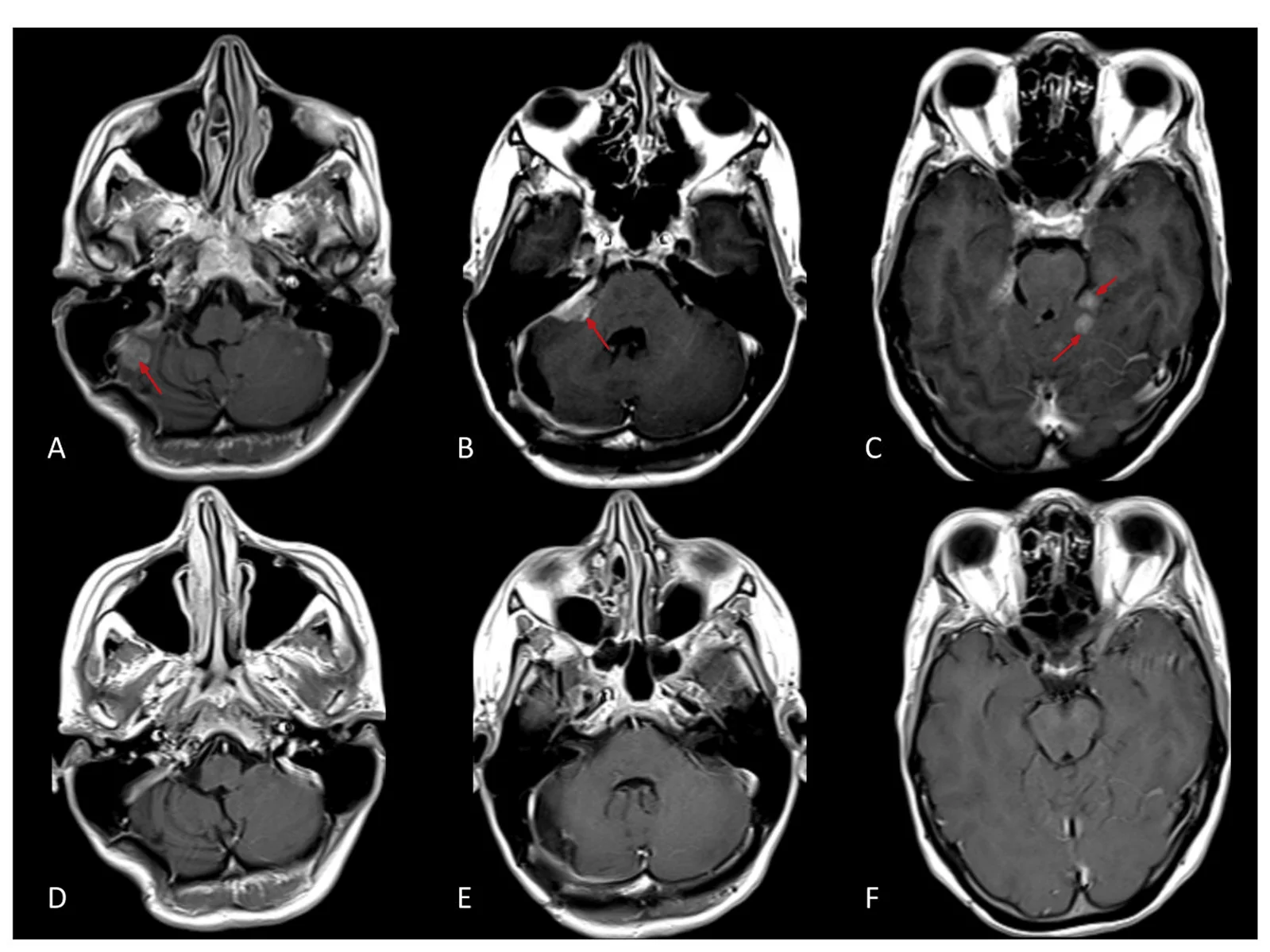
Complete intracranial response to trastuzumab deruxtecan Axial post-contrast T1-weighted MR images through the posterior fossa, displayed from inferior to superior levels. (A–C) Extra-axial enhancing metastatic lesions (red arrows): a right-sided lesion at the inferior cerebellar level, implanted in the right temporal recess and causing local mass effect (A); an enhancing extra-axial metastatic lesion at the level of the internal auditory canals, with a subtle dural tail and local mass effect (B); and two rounded enhancing extra-axial lesions along the superior cerebellar surface, adjacent to the free edge of the left tentorium (C). (D–F) Corresponding axial post-contrast images at the same anatomical levels demonstrate complete resolution of the lesions.

After 29 cycles of therapy as of April 2026, the patient remains clinically asymptomatic, tolerates treatment well, and shows no evidence of CNS disease on MRI, corresponding to a progression-free survival (PFS) exceeding 20 months.

## Discussion

This case illustrates the potential efficacy of T-DXd as first-line systemic therapy for symptomatic CNS-limited metastatic HER2-positive breast cancer involving predominantly extra-axial, dural-based, and leptomeningeal lesions. The patient experienced rapid neurological improvement within weeks, achieved complete radiologic resolution on MRI, and maintained a sustained response. The durable intracranial response and restoration of functional independence highlight the potential of T-DXd as a long-term therapeutic option in patients with CNS-limited disease.

Several features of this case merit specific emphasis. First, the pattern of CNS involvement was predominantly extra-axial and leptomeningeal rather than parenchymal, representing a distinct and clinically challenging subset that has been largely underrepresented in major clinical trials. Second, T-DXd was used in the first-line systemic metastatic setting, whereas the available evidence derives almost exclusively from pretreated patients with one or more prior lines of anti-HER2 therapy in the metastatic setting. These two factors together make this case particularly noteworthy.

The clinical outcomes observed in this patient are consistent with a growing body of clinical trial and real-world data. The pivotal phase III DESTINY-Breast03 trial, which led to the approval of T-DXd as second-line therapy for HER2-positive metastatic breast cancer, included a subgroup of patients with brain metastases. In this subgroup, T-DXd demonstrated an intracranial objective response rate (ORR) of 65.7% and a median PFS of 15 months [1,3]. It is important to note, however, that this subgroup included only patients with asymptomatic and clinically stable brain metastases, a substantial distinction from the symptomatic, active, and structurally complex CNS disease present in our patient.

The phase II TUXEDO-1 trial specifically enrolled patients with newly diagnosed or progressing active brain metastases from HER2-positive breast cancer who had no indication for immediate local therapy. In the primary outcome analysis, T-DXd achieved an intracranial response rate of 73.3%, including two complete responses [4]. Updated results published in 2024, at a median follow-up of 26.5 months, confirmed the durability of these responses, with a median PFS of 21 months and a median overall survival that had not yet been reached, alongside maintained quality of life and neurocognitive function [5]. These mature survival data from TUXEDO-1 are particularly relevant to our case and support the concept that T-DXd can provide prolonged intracranial disease control even in active CNS disease.

The DESTINY-Breast12 phase 3b/4 study is the largest prospective evaluation of T-DXd in patients with CNS metastases to date, enrolling 263 patients with stable or active brain metastases and 241 without brain metastases. The 12-month PFS rate in the brain metastases cohort was 61.6%, and the 12-month CNS PFS rate was 58.9%, confirming substantial and durable intracranial activity regardless of whether brain metastases were stable or active [6].

With respect to leptomeningeal disease specifically, evidence is more limited but supportive. The DEBBRAH trial evaluated T-DXd in patients with HER2-positive and HER2-low advanced breast cancer with CNS involvement, including a dedicated cohort (cohort 5) of seven patients with pathologically confirmed leptomeningeal carcinomatosis. This cohort had a median overall survival (OS) of 13.3 months, a median PFS of 8.9 months, and disease stabilization for ≥24 weeks in 71.4% of patients, with no unexpected safety signals [7]. While the small sample size limits generalizability, these results represent the most prospective evidence available for the leptomeningeal subgroup and are consistent with the durable response observed in our patient.

Real-world data further corroborate trial findings. The ROSET-BM study, a retrospective analysis including 104 patients with HER2-positive breast cancer and CNS disease, reported a median PFS of 14.6 months, an intracranial ORR of 62.7%, and a 24-month OS rate of 56%. Importantly, in the 19 patients with leptomeningeal carcinomatosis, the 12-month PFS and OS rates were 60.7% and 87.1%, respectively [8,9]. These real-world leptomeningeal outcomes are notably consistent with the durable response observed in our patient.

A retrospective pooled analysis of DESTINY-Breast01, -02, and -03 encompassing 148 patients with brain metastases treated with T-DXd further demonstrated intracranial ORRs of 45.2% in patients with treated or stable brain metastases and 45.5% in those with untreated or active brain metastases, with a median intracranial duration of response of 17.5 months in the active subgroup [10]. These data, together with an updated meta-analysis published in *Cancer Treatment Reviews* in 2025 [11], comprehensively support the intracranial activity of T-DXd across a spectrum of CNS disease.

The biological rationale for the intracranial activity of T-DXd is incompletely understood but likely multifactorial. The topoisomerase I inhibitor payload (deruxtecan) is a small membrane-permeable molecule that may diffuse across the BBB, and the high drug-to-antibody ratio of T-DXd (approximately 8:1) results in a high payload concentration per antibody molecule. Additionally, the disrupted vasculature of metastatic CNS lesions may increase BBB permeability, facilitating ADC extravasation. The bystander killing effect, whereby the membrane-permeable payload can enter and kill adjacent HER2-negative tumor cells, may be particularly relevant in the heterogeneous microenvironment of leptomeningeal and dural lesions [12].

This case also raises important questions about the optimal timing and sequencing of T-DXd in patients with CNS-limited disease. The first-line use of T-DXd in our patient, at a time when all trial evidence pertained to pretreated populations, merits discussion. The phase III DESTINY-Breast09 trial has since demonstrated superior PFS with first-line T-DXd plus pertuzumab over the standard THP regimen (40.7 vs. 26.9 months, with a hazard ratio of 0.56), resulting in FDA approval in December 2025 [13]. Patients with baseline brain metastases showed consistent benefit in this trial, lending coherence to our approach and highlighting the need for prospective studies specifically addressing first-line T-DXd in CNS-limited or CNS-predominant HER2-positive disease [14].

A further practical consideration is tolerability in this context. In our patient, T-DXd was well tolerated across 29 cycles with no treatment-limiting toxicity. Although T-DXd has demonstrated a generally manageable safety profile across clinical trials, ILD/pneumonitis remains an important treatment-specific risk, occurring in approximately 11-17% of patients in DESTINY-Breast03, DESTINY-Breast12, and DESTINY-Breast09, with most events being low grade [1,6,13]. These data highlight the favorable long-term tolerability observed in our patient while reinforcing the importance of continued monitoring during prolonged T-DXd treatment.

## Conclusions

This case demonstrates a rapid, complete, and durable intracranial response to first-line T-DXd in HER2-positive breast cancer with symptomatic CNS involvement, including leptomeningeal disease, accompanied by recovery of functional independence. The sustained response and favorable tolerability support the intracranial activity of T-DXd in this challenging and underrepresented population. Further prospective studies including patients with active and symptomatic CNS disease are warranted.
